# Zig, Zag, and ’Zyme: leveraging structural biology to engineer disease resistance

**DOI:** 10.1007/s42994-024-00152-w

**Published:** 2024-04-11

**Authors:** Alexander J. McClelland, Wenbo Ma

**Affiliations:** The Sainsbury Laboratory, Norwich Research Park, Norwich, NR4 7UH UK

**Keywords:** Plant immunity, Structural biology, Bioengineering, Cell wall-degrading enzymes, Receptor biology

## Abstract

Dynamic host–pathogen interactions determine whether disease will occur. Pathogen effector proteins are central players in such disease development. On one hand, they improve susceptibility by manipulating host targets; on the other hand, they can trigger immunity after recognition by host immune receptors. A major research direction in the study of molecular plant pathology is to understand effector-host interactions, which has informed the development and breeding of crops with enhanced disease resistance. Recent breakthroughs on experiment- and artificial intelligence-based structure analyses significantly accelerate the development of this research area. Importantly, the detailed molecular insight of effector–host interactions enables precise engineering to mitigate disease. Here, we highlight a recent study by Xiao et al., who describe the structure of an effector-receptor complex that consists of a fungal effector, with polygalacturonase (PG) activity, and a plant-derived polygalacturonase-inhibiting protein (PGIP). PGs weaken the plant cell wall and produce immune-suppressive oligogalacturonides (OGs) as a virulence mechanism; however, PGIPs directly bind to PGs and alter their enzymatic activity. When in a complex with PGIPs, PGs produce OG polymers with longer chains that can trigger immunity. Xiao et al. demonstrate that a PGIP creates a new active site tunnel, together with a PG, which favors the production of long-chain OGs. In this way, the PGIP essentially acts as both a PG receptor and enzymatic manipulator, converting virulence to defense activation. Taking a step forward, the authors used the PG-PGIP complex structure as a guide to generate PGIP variants with enhanced long-chain OG production, likely enabling further improved disease resistance. This study discovered a novel mechanism by which a plant receptor plays a dual role to activate immunity. It also demonstrates how fundamental knowledge, obtained through structural analyses, can be employed to guide the design of proteins with desired functions in agriculture.

The host–pathogen arms race has been described as a zig-zag process with step-wise co-evolution (Jones and Dangl 2006). While plants have evolved a sophisticated immune system to defend against pathogen invasion, pathogens secrete effector proteins to overcome host defense mechanisms and promote disease. As a counter-counter-defense, some effectors “trip the wire” and are recognized by immune receptors to activate immunity. According to their localizations in plant tissues, pathogen effectors are classified as apoplastic or cytoplasmic. Recognition of apoplastic effectors relies on membrane-bound receptor-like kinases or proteins (RLKs or RLPs), which often involves direct binding to the effector. Meanwhile, cytoplasmic effectors are recognized by intracellular NLR receptors that contain the conserved nucleotide-binding, leucine-rich repeat (NB-LRR) domain. Activation of these receptors may result from direct or indirect recognition of effectors. With indirect recognition, the receptor “guards” specific host proteins or processes that are manipulated by effectors and translates the detected abnormalities into a defense response (Spoel and Dong 2012; van der Hoorn and Kamoun 2008).

This interplay of defense and counter-defense is often centered on enzymes. For example, kinases are important components of immune signaling, and many pathogens produce kinase inhibitors to disrupt the signaling process (Zhou and Chai 2008). Protease inhibitors are also commonly produced by pathogens to overcome plant proteases having functions in defense (Godson and van der Hoorn 2021). Similarly, pathogens employ effectors with various enzymatic activities to manipulate the host. Some of the most important apoplastic effectors are cell wall-degrading enzymes (CWDEs). CWDEs are secreted by a wide range of microbial pathogens and insects (Lagaert et al. 2009). Because the cell wall is a crucial barrier to invading pathogens, CWDEs play a key role in aiding infection by weakening the cell wall of the host. In line with the zig-zag model, one counter-counter-defense mechanism plants have evolved to “guard” the cell wall is to deploy inhibitors of pathogen CWDEs.

A prominent example of CWDEs in the host–pathogen arms race involves pathogen polygalacturonases (PGs) and their plant-derived inhibitors, called PG-inhibiting proteins or PGIPs (De Lorenzo et al. 2001; Juge 2006; Kalunke et al. 2015; Lagaert et al. 2009). Interestingly, PGs have dual functions. In addition to disrupting cell wall integrity, pectin degradation by PGs leads to the accumulation of oligogalacturonides (OGs), which possess immunosuppressive functions and, thus, further promote virulence (Moerschbacher et al. 1999). PGs can be recognized by plant PGIPs, which contain a central LRR domain similar to those present in immune receptors (Di Matteo et al. 2006). In particular, RLPs directly bind to extracellular ligands through their LRR domain (Wan et al. 2019).

Unlike the RLPs, PGIPs lack a transmembrane domain and are not directly associated with the plasma membrane. In addition, although called “inhibitors”, PGIPs can alter the enzymatic activity of pathogen PGs without blocking pectin degradation in a way that the OGs produced have longer chains. Long-chain OGs with 10–15 degrees of polymerization act as signals that indicate cell wall damage and activate defense responses through wall-associated kinases (WAKs) (Brutus et al. 2010). As such, PGIPs confer disease resistance, via a mechanism that is rather unusual compared to other immune receptors and inhibitors in that they “hijack” the enzymatic activity of a pathogen virulence protein to produce immune-eliciting molecules. Although this observation has been made for over 30 years (Cervone et al. 1989), an explanation of how PGIPs alter the enzymatic activity of PGs remained elusive.

The recent publication “A plant mechanism of hijacking pathogen virulence factors to trigger innate immunity” by Xiao et al. (2024) in *Science* represents a landmark in elucidating the long sought-after mechanism by which PGIPs manipulate PGs to confer resistance (Xiao et al. 2024). Deploying a dynamic approach comprising structural biology, enzyme biochemistry, and functional *in planta* assays, Xiao et al. investigated the interaction between the common bean (*Phaseolus vulgaris*) PGIP2 (PvPGIP2) and the fungal pathogen *Fusarium phyllophilum* PG (FpPG). In vitro, they confirmed the formation of a PvPGIP2-FpPG complex and the propensity of the complex to produce longer OG polymers than FpPG alone using de-esterified pectin (polygalacturonic acid or PGA) as the substrate. Using pathogen infection and immune activation assays, they demonstrate that FpPG-PvPGIP2 + PGA treatment activates defense, *in planta*, likely by producing long-chain OGs*.* Conversely, FpPG + PGA, in the absence of PvPGIP2, produced shorter OGs that can suppress immunity triggered by pathogen-associated molecular patterns (PAMPs), such as chitin. Together, these results confirm that PvPGIP2 alters the enzymatic activity of FpPG and induces the accumulation of long-chain OGs, boosting plant immunity and mitigating pathogen infection (Xiao et al. 2024).

Excitingly, Xiao et al. solved a high-resolution structure of PvPGIP2 in complex with FpPG. Two regions of FpPG form interacting interfaces with PvPGIP2; however, PvPGIP2 does not directly block the active site of FpPG. Interaction with PvPGIP2 also did not change the overall conformation of FpPG. Instead, PvPGIP2 creates a new active site “tunnel” together with FpPG. Although PvPGIP2 alone does not bind to long-chain OGs, it increased the binding affinity of FpPG to long-chain OGs, but not short-chain OGs, potentially altering the enzymatic specificity. The PvPGIP2-FpPG complex structure revealed a cluster of positively charged residues in PvPGIP2 that forms part of the new active site tunnel. A mutant containing substitutions of these residues to non-charged amino acids exhibited reduced binding affinity to long-chain OGs, and the mutant, when combined with FpPG, resulted in the production of short-chain OGs, similar to FpPG alone. It is worth noting that these positively charged residues are outside of the FpPG-interacting interfaces, thereby decoupling the interaction and manipulation activities of PvPGIP2. Therefore, PvPGIP2 alters the active site of FpPG to induce immunity (Fig. 1) (Xiao et al. 2024).

**Fig. 1:**
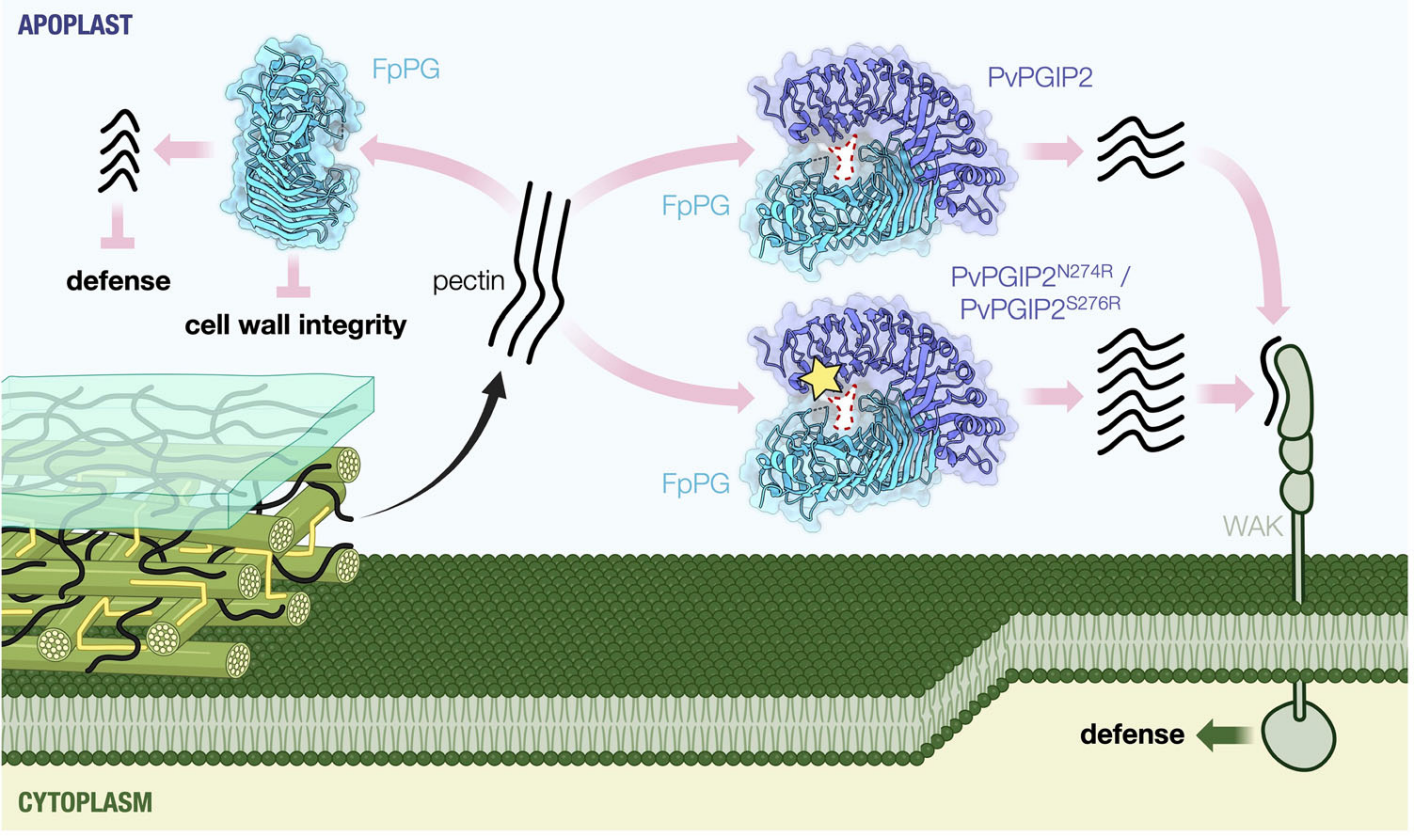
PvPGIP2 alters FpPG enzymatic activity to induce plant defense. Alone, FpPG degrades host cell wall pectin to weaken the cell wall and produce immunosuppressive oligogalacturonides. PvPGIP2, in complex with FpPG, induces the formation of a new active site tunnel (highlighted by the red-dashed circle), which favors the production of long-chain oligogalacturonides. Instead of suppressing defense, the long-chain oligogalacturonides activate immune signaling through plasma membrane-bound receptors (wall-associated kinases or WAKs). Detailed characterizations of the structure–function relationship of the PvPGIP2-FpPG complex guided the design of PvPGIP2 variants (PvPGIP2^N274R^ and PvPGIP2^S276R^, the residues are highlighted by the yellow star), which enable the enhanced production of long-chain oligogalacturonides. These modifications hold the promise of further elevating disease resistance. Figure created with BioRender.com

Given the crucial role of the positively charged cluster in PvPGIP2 in long-chain OG binding, the authors next tested whether mutating nearby residues to arginine, a positively charged amino acid, could further increase long-chain OG production. Indeed, two variants, PvPGIP2^N274R^ and PvPGIP2^S276R^, exhibited increased production of long-chain OGs compared to wildtype PvPGIP2 when combined with FpPG (Fig. 1). Lastly, using the PvPGIP2-FpPG complex structure as a reference, they showed that a predicted structural model of *Medicago truncatula* PGIP1 (MtPGIP1) contained variant residues at the FpPG interaction interface. Consistent with this sequence variation, MtPGIP1 does not interact with FpPG, in vitro. Substitution of these residues in MtPGIP1 into the structurally equivalent ones in PvPGIP2 enabled FpPG binding and the production of long-chain OGs (Xiao et al. 2024). These experiments showcase how new-to-nature variants of PGIPs, with improved activities, can be generated based on structural information.

This study represents a significant advancement in our understanding of the host–pathogen arms race by shedding new light on a unique effector–receptor interaction. Inhibitors are widely used by both hosts and pathogens to defeat specific enzymatic activities in their counterpart. Often, inhibitors directly bind to the active sites to block or diminish the enzymatic activities. This has been well established for protease inhibitors. For example, many pathogens secrete effectors to inhibit host papain-like cysteine proteases (PLCPs) as an important virulence mechanism to promote infection and disease progression (Clark et al. 2018; Clark et al. 2020; Godson and van der Hoorn 2021; Misas-Villamil et al. 2016). In some cases, direct inhibition of key enzymatic activities can be “guarded” by immune receptors to trigger immunity. For example, the membrane-bound RLP Cf-2 from tomato recognizes inhibition of the PLCP Rcr3 by an apoplastic effector, Avr2, from the fungal pathogen *Cladosporium fulvum* (Rooney et al. 2005). What is unusual about PGIP-PG interaction is that plant PGIPs combine these principals together to act both as a receptor of PGs and a manipulator of the PG enzymatic activity to induce defense in tandem with WAKs, thereby “guarding” cell wall integrity (Fig. 1).

Although evidence of defense mechanisms involving “hijacking” of a specific activity of the pathogen has just emerged, similar tactics have been observed from pathogens as a robust virulence strategy. A most recent example came from the oomycete pathogen *Phytophthora*, which employs effectors containing “LWY” tandem repeat units to promote infection. Several LWY effectors are able to form functional holoenzymes with the core enzyme of the host protein phosphatase 2A (PP2A) (Li et al. 2023). The crystal structure of the effector-PP2A complex revealed a specific combination of two LWY units that confer PP2A interaction. This PP2A-interacting module is located at the N-terminus of the effectors, whereas their C-terminal LWY units are responsible for recruiting various phosphoproteins to the holoenzyme for dephosphorylation. As such, these LWY effectors hijack a major host protein phosphatase to regulate cellular functions. These recent publications demonstrate that both plants and pathogens have co-evolved “hijacking” mechanisms for their own benefit.

PGIPs have garnered a lot of interest in the development of disease resistant crops. PGIP-based resistance could have far-reaching effects in agriculture, given the prevalence of PGs and PGIPs across pathogens and plants, respectively. PGIPs are ubiquitous in the plant kingdom and have been demonstrated to increase resistance to fungal, oomycete, and bacterial pathogens. (De Lorenzo et al. 2001; Juge 2006; Kalunke et al. 2015; Lagaert et al. 2009). Structural information of the PGIP-PG complex unlocks exciting opportunities to engineer resistance in a targeted, precise manner. For example, the binding capacity of PGIPs to pathogen-produced PGs can be improved. PGs may carry variations from host–pathogen co-evolution, thereby escaping recognition by PGIPs. The interaction interface can be designed to broaden the binding capacity and inhibition spectrum of PGIPs. Moreover, PGIPs may be engineered for more superior PG manipulating activity, such as by modifying the active site tunnel (Fig. 1), elevating resistance efficiency.

Related to these exciting possibilities, an interesting avenue of future research would be to dive deeper into the natural structural and sequence variations that exist in both PGIPs and PGs, which will expand our potential to introduce effective PG monitoring in elite crop cultivars. This analysis will also inform how PGs may evolve to overcome PGIP manipulation, and if PGIPs have concurrently adapted novel PG-binding mechanisms to retain their defense function. Recently, the PGIP-inactivating effector PINE1, from necrotrophic fungi, was shown to block PGIP functions, essentially acting as a pathogen inhibitor of a plant inhibitor of a pathogen effector (Wei et al. 2022). A future area of research could explore the PINE1-PGIP interaction interface to engineer PINE1-insensitive PGIPs. Lastly, plants have evolved independent mechanisms to trigger defense upon the perception of PGs and other CWDEs. For example, a conserved 9-amino-acid fragment from fungal PGs is recognized by a canonical membrane-bound receptor RLP42 in *Arabidopsis thaliana*, thus triggering defense (Zhang et al. 2021). Further, the *Phytophthora sojae* CWDE XEG1 is inhibited by the RLP RXEG1, which in turn forms a complex with the co-receptor BAK1 to activate defense (Sun et al. 2022). Such structural insights may be incorporated to engineer novel inhibitors of CWDEs, including PGs.

Xiao et al. demonstrated the power of engineering designer proteins to improve desired activities, via a structure-guided approach. Given the abundance of experimentally validated protein structures, the increasingly accessible nature of protein complex structure predictions (Evans et al. 2021), and the emergence of de novo peptide design tools (Watson et al. 2023), our ability to engineer designer crops is steadily improving. Plant immune receptors are particularly enticing candidates for bioengineering to enhance disease resistance (Cadiou et al. 2023; Contreras et al. 2023a). For example, the crystal structure of the NLR receptor Pikp integrated heavy metal-associated (HMA) domain in complex with the rice blast fungal effector AVR-Pik guided the generation of a Pikp mutant that can recognize previously unrecognized AVR-Pik effectors (De la Concepcion et al. 2019). In another example, structure-guided engineering of the helper NLR, NRC2, blocked its inhibition by a nematode effector, while retaining its immune functions (Contreras et al. 2023b). The emergence of artificial intelligence further facilitates protein engineering by offering guidance through in silico protein structure prediction. In a recent study, AlphaFold multimer was used to identify interacting residues between the tomato cysteine protease Pip1 and EpiC2B, a protease-inhibiting effector from the potato late blight pathogen *Phytophthora infestans*. Mutagenesis of two targeted amino acids in Pip1 rendered it insensitive to EpiC2B inhibition, and this engineered Pip1 had enhanced resistance to *P. infestans* (Schuster et al. 2024).

We are unmistakably in the midst of a structure-guided engineering boom. Structural characterizations have played a pivotal role in the advancement of our fundamental understanding of microbial pathogenesis and plant immunity. We expect to witness rapid development in this area, and ultimately, the application of this knowledge to improve crop yield.
